# Oligodendrocytes Do Not Export NAA-Derived Aspartate In Vitro

**DOI:** 10.1007/s11064-016-1985-y

**Published:** 2016-07-09

**Authors:** Ana I. Amaral, Mussie Ghezu Hadera, Mark Kotter, Ursula Sonnewald

**Affiliations:** 1 Wellcome Trust-Medical Research Council Cambridge Stem Cell Institute, Anne McLaren Laboratory and Department of Clinical Neurosciences, University of Cambridge, West Forvie Building, Robinson Way, Cambridge, CB2 0SZ UK; 2 Department of Pharmacy, College of Health Sciences, Mekelle University, Tigray, Ethiopia; 3 Department of Neuroscience, Faculty of Medicine, Norwegian University of Science and Technology (NTNU), PO Box 8905, MTFS, 7491 Trondheim, Norway; 4 Department of Drug Design and Pharmacology, Faculty of Health and Medical Sciences, University of Copenhagen, Copenhagen, 2100 Denmark

**Keywords:** Brain cells, Glia, Energy metabolism, Glucose, Aspartate, Acetate

## Abstract

Oligodendroglial cells are known to de-acetylate the *N*-acetylaspartate (NAA) synthesized and released by neurons and use it for lipid synthesis. However, the role of NAA regarding their intermediary metabolism remains poorly understood. Two hypotheses were proposed regarding the fate of aspartate after being released by de-acetylation: (1) aspartate is metabolized in the mitochondria of oligodendrocyte lineage cells; (2) aspartate is released to the medium. We report here that aspartoacylase mRNA expression increases when primary rat oligodendrocyte progenitor cells (OPCs) differentiate into mature cells in culture. Moreover, characterising metabolic functions of acetyl coenzyme A and aspartate from NAA catabolism in mature oligodendrocyte cultures after 5 days using isotope-labelled glucose after 5-days of differentiation we found evidence of extensive NAA metabolism. Incubation with [1,6-^13^C]glucose followed by gas chromatography–mass spectrometry and high performance liquid chromatography analyses of cell extracts and media in the presence and absence of NAA established that the acetate moiety produced by hydrolysis of NAA does not enter mitochondrial metabolism in the form of acetyl coenzyme A. We also resolved the controversy concerning the possible release of aspartate to the medium: aspartate is not released to the medium by oligodendrocytes in amounts detectable by our methods. Therefore we propose that: aspartate released from NAA joins the cytosolic aspartate pool rapidly and takes part in the malate–aspartate shuttle, which transports reducing equivalents from glycolysis into the mitochondria for ATP production and enters the tricarboxylic acid cycle at a slow rate.

## Introduction

The brain is an organ with exceptionally high energy demands and relies on an uninterrupted supply of substrates for oxidative phosphorylation in mitochondria. Around 25 % of the body’s total glucose budget is spent on processes in the brain, including the generation of action potentials and synaptic transmission [1]. Glucose-derived energy is therefore of utmost importance for maintaining physiological function of the brain. In contrast, many neurodegenerative diseases including Alzheimer’s disease [2] are associated with compromised glucose metabolism and markers of low energy status. Quantification of *N*-acetylaspartate (NAA) has often been used to assess the metabolic integrity of neurons [3, 4]. NAA can be detected by ^1^H-magnetic resonance spectroscopy, and has been applied as a non-invasive quantitative method for detecting progression, recovery, and remission in an ever-increasing catalogue of disorders of the brain [5]. However, the fundamental role of NAA in the brain remains elusive and the available evidence for its function is limited to a role in providing acetyl groups for lipid synthesis [6].

NAA is amongst the most abundant amino acid derivatives in the brain and is synthesized from aspartate and acetyl coenzyme A (CoA) by aspartate *N*-acetyltransferase [7]. After birth, NAA content in the brain is subject to a rapid increase to reach concentrations of 5 to 10 mM [8, 9], being especially concentrated in gray matter-rich regions [9–12]. NAA synthesis is dependent on mitochondrial integrity [7, 13] and fluctuations in concentration can occur in parallel with changes in adenosine triphosphate (ATP) [14], suggesting an intimate relationship with metabolic energy.

NAA is produced by and released from neurons. These depend on the supply of precursor molecules provided by astrocytes to synthesize the aspartate necessary for NAA production [15, 16]. NAA is metabolized in oligodendrocytes, which contain aspartoacylase (ASPA) [17–19], the only known NAA-catabolizing enzyme in the brain. The importance of ASPA to myelination is highlighted by the severely dys-myelinated phenotype of the inherited human paediatric leukodystrophy, Canavan disease (CD), which results from the loss of ASPA function. The abnormally high levels of NAA in CD are in contrast to abnormally low levels of NAA that typically are seen in practically all other neurodegenerative diseases. Considering the importance of NAA as a prognostic marker of metabolic function across a wide pathological range the view that NAA solely acts as a shuttle for acetyl groups during lipid synthesis may therefore be insufficient. Multiple roles have been suggested for NAA such as: being an osmolyte that acts as a “molecular water pump” to remove metabolically produced water from neurons [20, 21]; in addition to moving acetate groups across the mitochondrial membrane system, NAA also acts by moving nitrogen groups to the cytoplasm [22]; NAA is involved in facilitating glutamine/glutamate oxidation in neuronal mitochondria, while bypassing the glutamate dehydrogenase reaction and therefore avoiding ammonia production [23]; being a storage and transport form of acetate [24]. Other proposed roles include the involvement in histone and protein acetylation reactions [25]; altering metabolism (aerobic glycolysis and Warburg effect) in cancer cells [26, 27] and, more recently, it was linked to neuronal differentiation [28].

In order to investigate the role of NAA in intermediary metabolism, we incubated cultures enriched for mature oligodendrocytes in medium containing [1,6-^13^C]glucose for 8 and 24 h in the presence and absence of NAA and analysed cell extracts and media using gas chromatography–mass spectrometry (GC–MS) and high performance liquid chromatography (HPLC). We found that the acetyl CoA produced by hydrolysis of NAA does not enter mitochondrial metabolism. We propose that the aspartate joins the aspartate pool active in the malate–aspartate shuttle and we established that aspartate is not released to the medium in amounts detectable by our methods.

## Materials and Methods

### Materials

Cell culture reagents were purchased from Sigma (Dorset, UK)—Dulbecco’s modified Eagle’s medium (DMEM), minimum essential medium Eagle (MEM), l-glutamine, poly-l-lysine (PLL), papain, NAA—or Life Technologies (Paisley, UK)—fetal bovine serum (FBS), penicillin–streptomycin (pen–strep), trypsin–EDTA, phosphate buffered saline (PBS). ^13^C-labelled compounds were obtained from Cambridge Isotope Laboratories, MA, USA. The mass spectrometry derivatization reagents MTBSTFA (*N*-methyl-*N*-(*tert*-butyldimethylsilyl) trifluoroacetamide), MSTFA (*N*-methyl-*N*-(trimethylsilyl) trifluoroacetamide) and the t-BDMS-Cl (*tert*-butyldimethylchlorosilane) were purchased from Regis Technologies, Inc. (Morton Grove, IL, USA). Recombinant human PDGF-AA and Recombinant human FGF-basic were purchased from PeproTech (Rocky Hill, NJ). All other chemicals were of the purest grade available from Sigma (Dorset, UK).

### Primary Cultures of Rat Oligodendrocytes

Primary mixed glia cultures were isolated from neonatal Sprague Dawley rat (postnatal day 0–2) forebrains following established protocols [29]. Briefly, pups were euthanized according to “Schedule 1” regulations from the Home Office Animal Procedures Committee UK. Cells were cultured for 10–15 days in DMEM supplemented with 10 % FBS, 1 % pen-strep and 4 mM glutamine, and kept under a humidified atmosphere at 37 °C and 7 % CO_2_. Oligodendrocyte precursor cells (OPCs) were subsequently isolated using a step-based shake-off protocol and cultured in Sato’s medium on PLL-coated plates [29]. To obtain immature OPCs, cells were cultured for 1 day in Sato’s medium supplemented with human PDGF-AA and human recombinant FGF at 10 ng/ml. To induce differentiation, OPCs were cultured in Sato’s medium supplemented with 0.5 % fetal calf serum (FCS) for 1 or 5 days. The cell culture medium was replaced by fresh medium at day two of differentiation. For all experiments only cultures with >93 % purity (determined based on O4 immunostaining) were used [30].

### Quantitative Reverse Transcriptase PCR

To determine the mRNA levels of ASPA during OPC differentiation, cells were cultured either for 1 day in proliferation medium, or for 1 or 5 days in differentiation medium. Total RNA was extracted using the RNeasy Mini Kit (Qiagen, Manchester, UK). cDNA was synthesized from 20 ng RNA per sample using the Maxima First Strand cDNA Synthesis Kit (Thermo Scientific, Waltham, MA USA). Quantitative PCR (qPCR) was conducted as previously outlined [29] on an Applied Biosystems 7500HT Fast Real-time PCR system. The primers used were: ASPA F-AGACGTGGCTGCTGTTATCC; ASPA R-GATCTCCAGGGTGCAATGGT; beta actin F-CATGGCATTGTGATGGACT; beta actin R-ACGGATGTCAACGTCACACT. Values are represented as ASPA/beta actin ratios. Measurements were made on 8–11 samples obtained from three independently generated cultures.

### Incubations with ^13^C Labelled Glucose and NAA

OPCs isolated from mixed glia cultures were cultured in 6 well plates at a cell density of 4 × 10^5^ cells/well and allowed to differentiate for 5 days (see above for details). Prior to incubation, cells were washed once with PBS and incubated with 2 ml Sato’s medium prepared from a glucose, glutamine and pyruvate-free DMEM (Sigma D5030, Dorset, UK) supplemented with 0.5 % FCS and 2 mM [1,6-^13^C]glucose, 2 mM glutamine and 2 mM NAA (controls did not have NAA in the incubation medium) for 8 and 24 h. Samples of medium were collected before and after the incubation period and subsequently analysed by mass spectrometry. To stop the incubation, cells were washed twice with cold PBS and the intracellular metabolites extracted with 70 % ethanol [31]. Experiments were performed in four independently generated cultures with, at least, two replicate wells per condition.

### Glucose and Lactate Analyses

Glucose and lactate levels in the cell culture medium were analysed at the Core Biochemical Assay Laboratory, Clinical Biochemistry, Addenbrooke’s Hospital using automated assays on a Siemens Dimension RxL analyser. The rate of glucose and lactate net change relative to cells over time (µmol/10^6^ cells/24 h) was calculated by subtracting the value measured at the end of the experiment (T = 24 h) from the one measured in a sample of medium collected at the onset of the incubation, and dividing the resulting value by the amount of cells in each experiment, multiplied by the experimental volume (2 ml). The cell number considered in the calculations was the cell number at plating since oligodendrocytes do not proliferate. Analyses were performed on 9–12 samples, which derived from four independently generated cultures.

### High Performance Liquid Chromatography (HPLC)

HPLC was used to quantify the total amounts of amino acids in samples of cell extracts. Samples were lyophilized and re-suspended in 0.01 M HCl and subsequently derivatised with *o*-Phthaldialdehyde [32] using an automated method prior to injection into the HPLC column. Amino acid concentrations were determined by comparison to a calibration curve of standard solutions of amino acids run after every 12 samples. Analyses were performed on 6–11 samples derived from three independently generated cultures. For details see Amaral et al. [32].

### Gas Chromatography–Mass Spectrometry (GC–MS)

For analysis of percent enrichment with ^13^C in lactate, amino acids (aspartate, glutamate and glutamine) and TCA cycle intermediate (citrate) after incubation with [1,6-^13^C]glucose ± NAA, cell extracts and samples of medium were lyophilized and re-suspended in 0.01 M HCl. Derivatisation with MTBSTFA in the presence of 1 % *t*-BDMS-Cl [33] was performed as described in Amaral et al. [30]. The samples were analysed on an Agilent 6890 gas chromatograph connected to an Agilent 5975B mass spectrometer (Agilent Technologies, Palo Alto, CA). The parent ion (M) and atom percent excess for one ^13^C atom (M + 1) values for lactate, citrate, glutamate, glutamine and aspartate were calculated from the GC–MS data using the MassHunter software supplied by Agilent (Agilent Technologies, Palo Alto, CA) and correcting for the naturally abundant ^13^C by using non-enriched standards [34]. Analyses were performed on 6–12 samples of medium and 8–11 samples of extracts derived from four independently generated cultures.

### Statistical Analysis

Statistical analysis was conducted using unpaired two-tailed Student *t* tests (confidence interval = 95 %) to compare the effect of NAA on the intracellular amounts of amino acids, on enrichment in ^13^C in extracellular and intracellular metabolites and on the glucose consumption and lactate release rates. To evaluate differences in the expression of ASPA mRNA levels at different stages of OPC maturation in culture, a one-way ANOVA (alpha = 0.05) followed by Tukey’s multiple comparison test was performed.

## Results

**Fig. 1:**
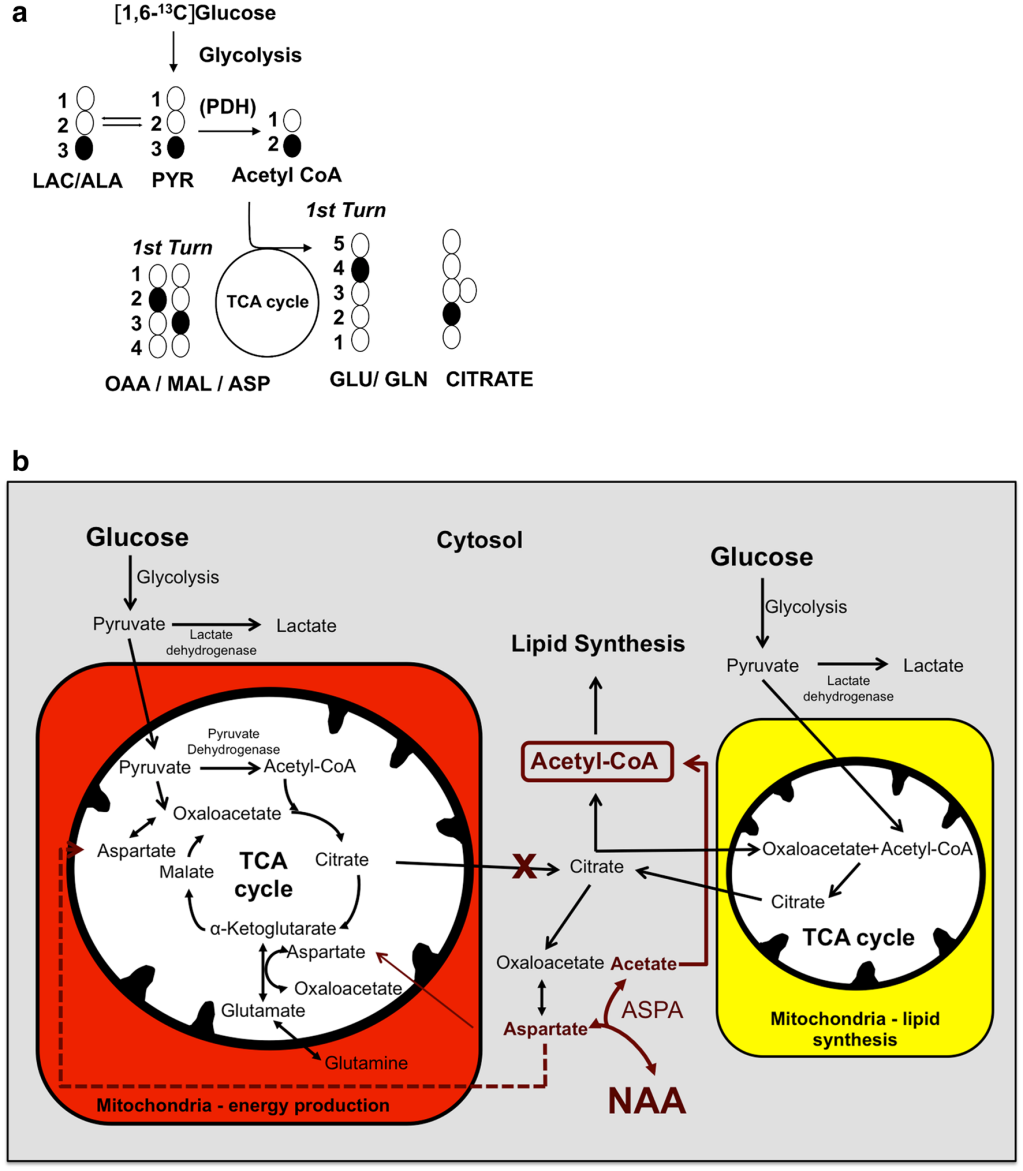
**a** ^13^C glucose labelling patterns in oligodendrocytes. [1,6-^13^C]Glucose is taken up by oligodendrocytes and converted to 2 molecules of [3-^13^C]pyruvate via glycolysis. Lactate dehydrogenase converts [3-^13^C]pyruvate to [3-^13^C]lactate (LAC) and alanine aminotransferase to alanine (ALA). Alternatively, [3-^13^C]pyruvate can enter the mitochondria to be metabolized to [2-^13^C]acetyl CoA which can enter the TCA cycle. The subsequent TCA cycle metabolites such as: citrate, malate (MAL) and oxaloacetate (OAA) and amino acids such as aspartate (ASP), glutamate (GLU) and glutamine (GLN), will be labelled with one ^13^C atom, resulting in an increase in the parent ion M mass to M + 1, which can be detected by GC–MS. **b** Schematic presentation of glucose metabolism in oligodendrocytes in the presence of NAA. We propose that at least two distinct TCA cycles exist: (1) one TCA cycle (depicted on the *left*) in which most of the energy is produced and in which only a fraction of citrate leaves the cycle and contributes to lipid synthesis. This “energy producing” TCA cycle is halted by the presence of NAA, which results in increased labelling of e.g. glutamate and glutamine and, to a lesser degree, citrate. The aspartate derived from NAA does not enter into this (1) TCA cycle during the initial phase (up to 8 h), since this would reduce labelling of glutamate and glutamine. In contrast, we found that labelling of glutamate and glutamine increased but labelling of aspartate did not increase. These findings indicate that the aspartate derived from NAA enters the malate–aspartate shuttle and dilutes aspartate labelling. (2) A second TCA cycle is implied by the increased ^13^C labelling of citrate in the medium, which occurred much more extensively than labelling of other metabolites after 24 h incubation with [1,6-^13^C]glucose. The citrate producing TCA cycle for lipid synthesis was not affected by the presence of NAA as the unchanged labelling of the major pool of citrate suggests

To investigate how NAA is metabolised by mature oligodendrocytes, we incubated primary OPC cultures maintained in differentiation medium for 5 days with [1,6-^13^C]glucose in the presence or absence of NAA. By assessing differences in metabolite enrichment in ^13^C, this strategy enabled us to study aspartate and acetate, both metabolic products of NAA, in metabolic pathways of glucose. For details on ^13^C labelling Fig. 1a. [1,6-^13^C]Glucose is taken up by oligodendrocytes and converted to 2 molecules of [3-^13^C]pyruvate via glycolysis. Lactate dehydrogenase converts [3-^13^C]pyruvate to [3-^13^C]lactate or [3-^13^C]pyruvate, which can enter the mitochondria to be further metabolized to [2-^13^C]acetyl CoA and enter the TCA cycle. The subsequent TCA cycle metabolites and amino acids derived from oxaloacetate and α-ketoglutarate, aspartate and glutamate, respectively, will be labelled with one ^13^C atom, resulting in mass M + 1. When a labelled acetyl CoA molecule condenses with a labelled oxaloacetate molecule the subsequent metabolites will contain two ^13^C atoms which will result in mass M + 2. Apart from its role in the TCA cycle, citrate is used to funnel acetyl CoA units into lipid synthesis. Figure 1b provides an overview of the metabolic pathways assessed in the present study. We postulated that oligodendrocytes possess distinct mitochondria or TCA cycles that are specialised for lipid synthesis and others that prioritise energy and amino acid production (Fig. 1b). When NAA enters the cell it is hydrolyzed into acetyl CoA and aspartate by ASPA. Whereas acetate is used for lipid synthesis (Fig. 1b), aspartate can then participate in the malate–aspartate shuttle (for details see [32]) or/and enter the TCA cycle after conversion to oxaloacetate.

**Fig. 2:**
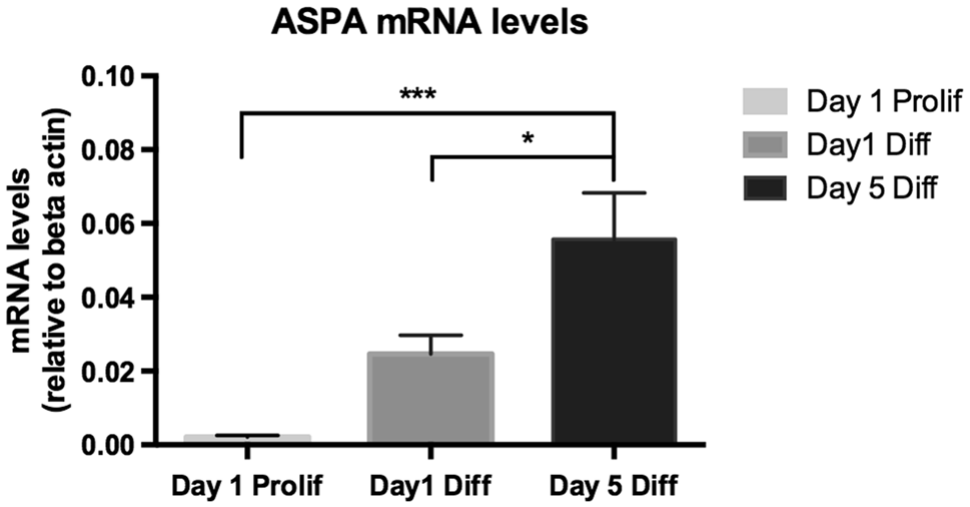
Aspartoacylase (ASPA) mRNA levels during OPC differentiation in vitro. Cells were cultured either for 1 day in proliferation medium, or for 1 or 5 days in differentiation medium. Values are represented as ASPA/beta actin ratios. Measurements were made on 8–12 samples obtained from three independently generated cultures (for details see “Methods” section)

In order to verify that the oligodendrocyte cultures used in the present study expressed the enzyme responsible for NAA hydrolysis, we quantified the ASPA mRNA levels. This demonstrated an increase in ASPA mRNA expression from day 1 to day 5 of differentiation (Fig. 2). All subsequent experiments were performed on 5-day-old cultures.

**Fig. 3:**
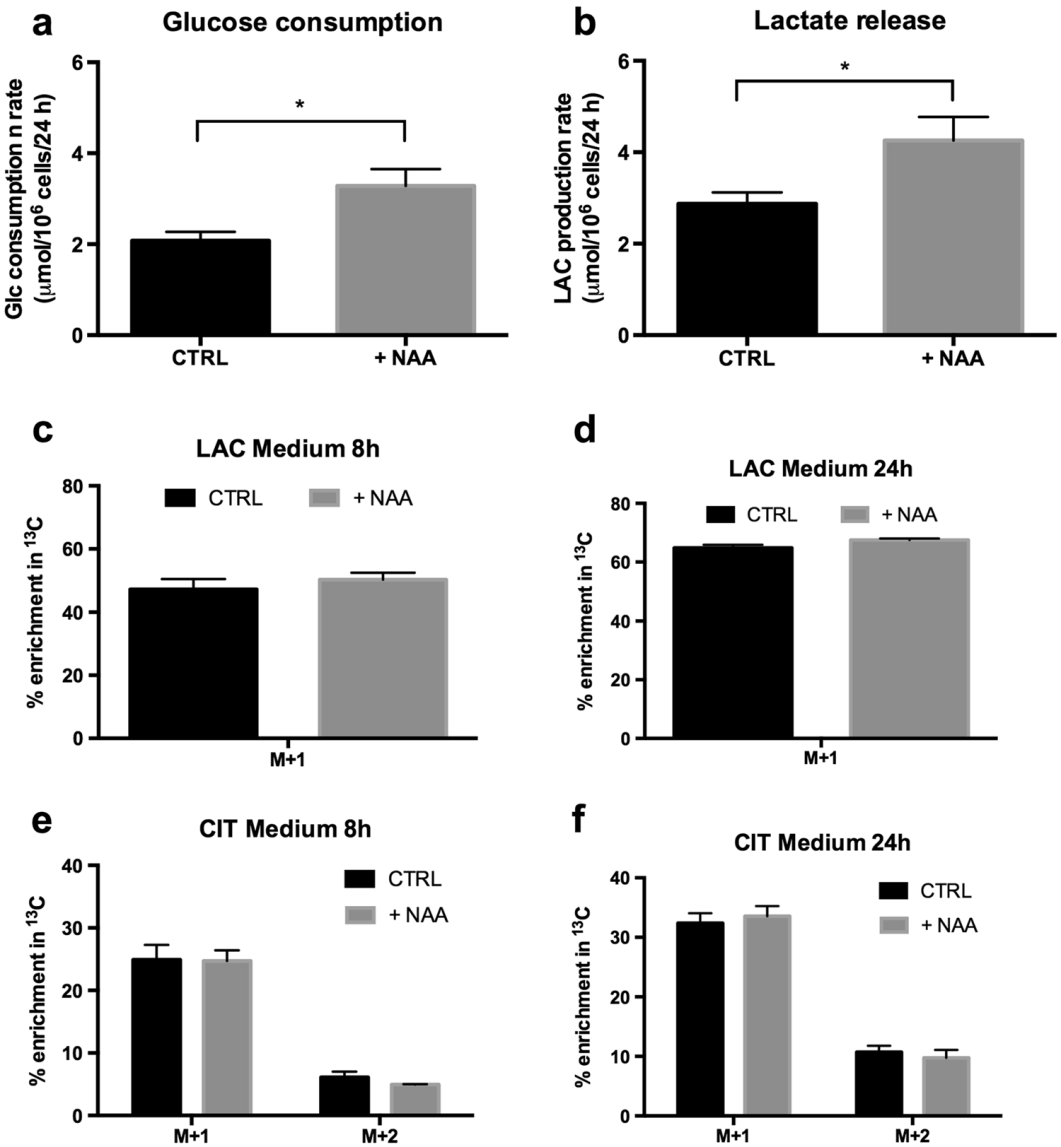
Effect of NAA on glucose consumption, lactate release, and enrichment with ^13^C of extracellular metabolites in mature oligodendrocyte cultures. Oligodendrocytes were incubated in medium containing [1,6-^13^C]glucose, 2 mM glutamine and 2 mM *N*-acetyl aspartate (NAA). Controls did not have NAA in the incubation medium. Samples of medium were collected and subsequently analysed by gas chromatography–mass spectrometry. % ^13^C enrichment above natural abundance is given for lactate (LAC) and citrate (CIT) after 8 or 24 h of incubation. Experiments were performed on 6–12 samples, which derived from four independently generated cultures. Glucose consumption and lactate release were measured in 9–12 samples obtained from four independently generated cultures after 24 h of incubation (for details see “Methods” section)

Cells were incubated in medium containing [1,6-^13^C]glucose for 8 and 24 h in the presence and absence of NAA. The presence of NAA increased glucose consumption and lactate production (Fig. 3a, b). The theoretical maximum enrichment of lactate from [1,6-^13^C]glucose is 100 %. However, after 8 h only approximately 50 % of lactate was ^13^C labelled, reaching approximately 65 % after 24 h (Fig. 3c, d). The addition of NAA did not affect ^13^C enrichment in lactate (Fig. 3c, d). Similarly, ^13^C-enriched citrate was released to the medium, but its enrichment was not affected by the addition of NAA (Fig. 3e, f).

**Fig. 4:**
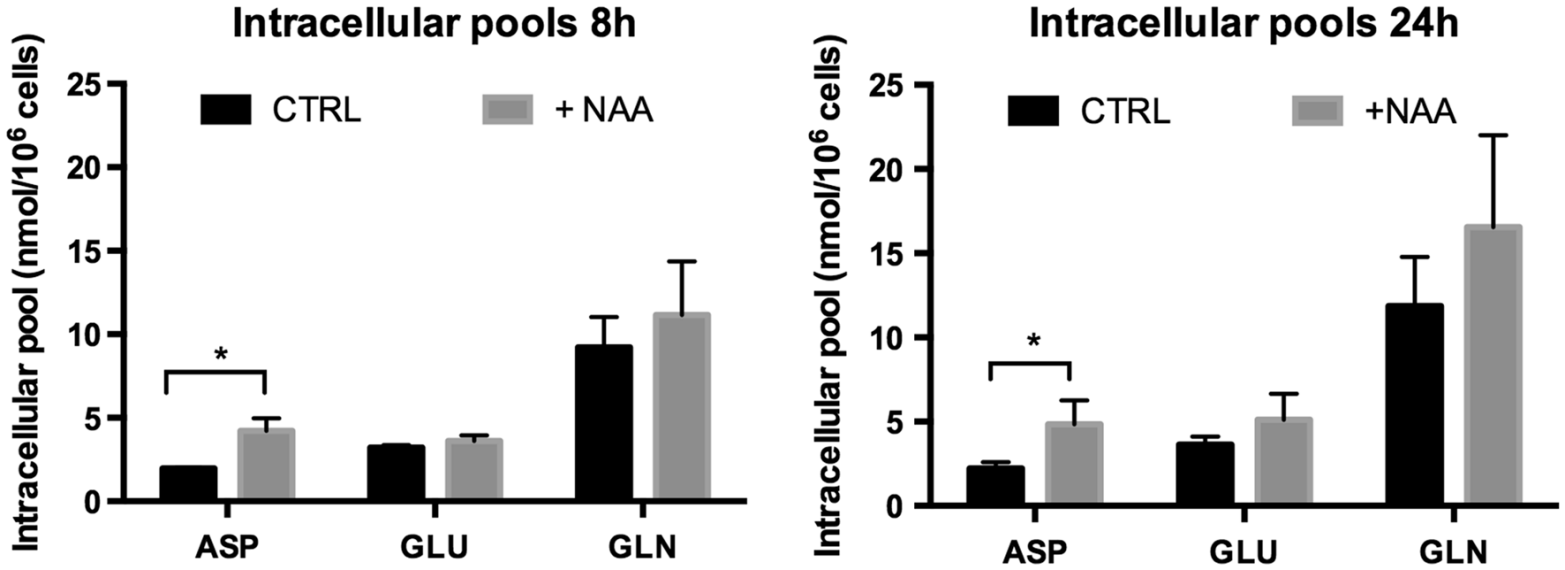
Effect of NAA on the intracellular levels of amino acids in mature oligodendrocyte cultures. HPLC was used to quantify the amounts of aspartate (ASP), glutamate (GLU) and glutamine (GLN) in samples of cell extracts of oligodendrocytes cultured for 5 days in differentiation medium and incubated for 24 h in the presence or absence of 2 mM *N*-acetyl aspartate (NAA). Analyses were performed on 6–11 samples, which derived from three independently generated cultures. Amounts are given in nmol/10^6^ cells (for details see “Methods” section)

The intracellular amounts of aspartate, glutamine and glutamate after 8 and 24 h incubation are provided in Fig. 4. Aspartate was increased in the presence of NAA whereas glutamine and glutamate were not (Fig. 4).

**Fig. 5:**
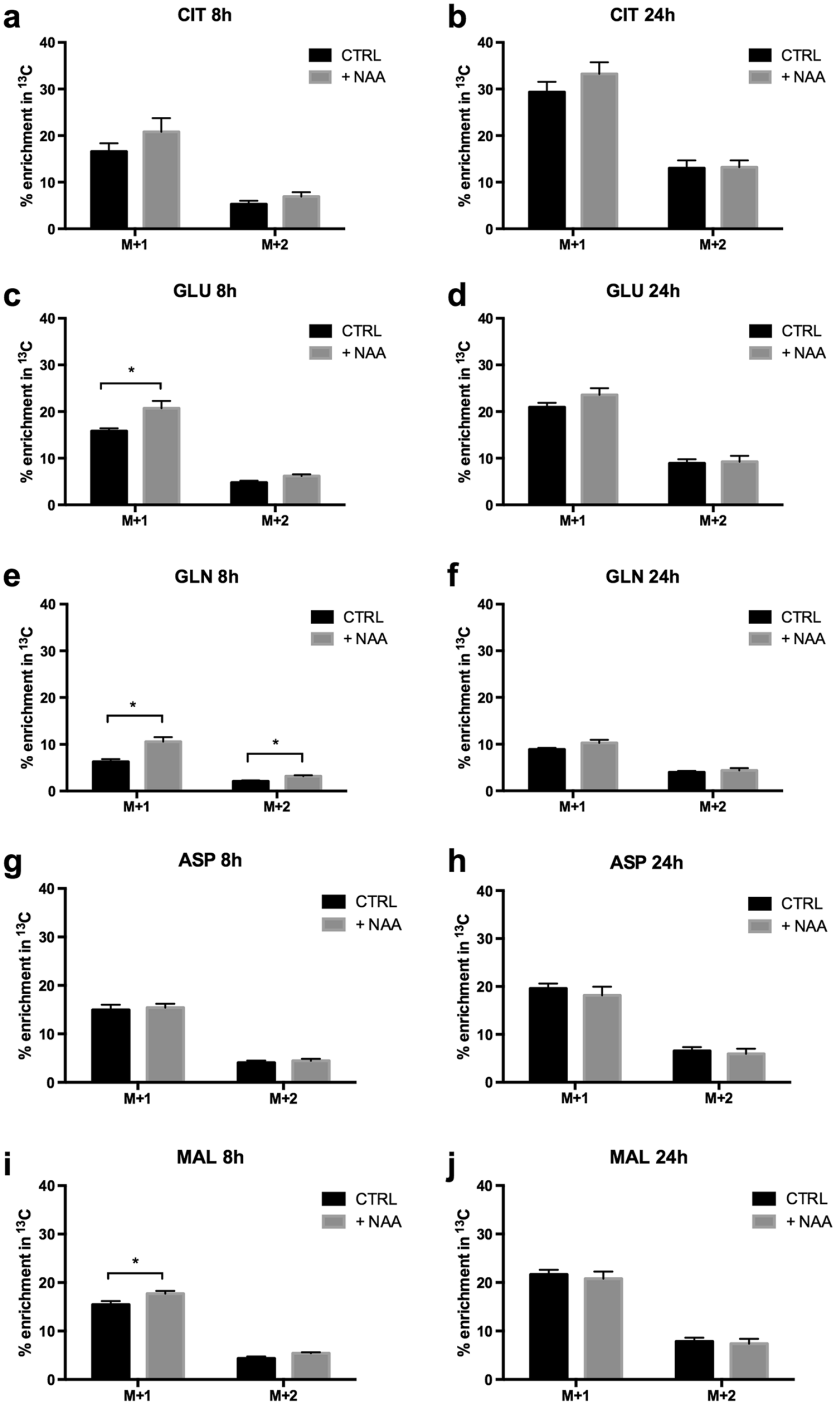
Effect of NAA on [1,6-^13^C]glucose-derived enrichment with ^13^C in intracellular metabolites of mature oligodendrocytes. Oligodendrocytes differentiated for 5 days were incubated in medium containing [1,6-^13^C]glucose, 2 mM glutamine and 2 mM *N*-acetyl aspartate (NAA). Controls did not have NAA in the incubation medium. Cell extracts were collected and subsequently analysed by gas chromatography–mass spectrometry. % ^13^C enrichment above natural abundance is given for aspartate (ASP), glutamate (GLU), glutamine (GLN), citrate (CIT) and malate (MAL) after 8 or 24 h of incubation. Experiments were performed on 8–11 samples, which derived from four independently generated cultures

Analysis of ^13^C incorporation from [1,6-^13^C]glucose into intracellular metabolites was conducted after 8 and 24 h in the absence or presence of NAA (Fig. 5). No differences were detected in ^13^C enrichment in any metabolite analysed after 24 h (Fig. 5) and aspartate and citrate were also not affected at 8 h (Fig. 5). All other metabolites displayed an increase in ^13^C enrichment after 8 h incubation with [1,6-^13^C]glucose and NAA as compared to cultures that did not receive NAA (Fig. 5).

## Discussion

Glucose metabolism in neurons and astrocytes has been studied extensively and much is known about the interaction between these two cell types in the brain [35]. However, until very recently oligodendrocytes have not been studied in this context [30, 35–38].

It was evident that the metabolism of *N*-acetylaspartylglutamate (NAAG) has a tri-cellular compartmentation, however, this was not clear in regard to NAA [38–40]. Both NAA and NAAG are synthesized by neurons in the brain. Neurons release both NAA and NAAG to the extracellular space upon stimulation, where astrocytes, the target cells for NAAG, hydrolyse it, releasing NAA, which is subsequently catabolised in oligodendrocytes. It has been proposed that oligodendrocytes release the aspartate obtained from NAA for recycling to neurons [39]. This hypothesis assumes that aspartate produced from NAA is passed from oligodendrocytes back to neurons to be reutilized for re-synthesis of NAA and NAAG. This exchange of metabolites would be in analogy with the well-established glutamine–glutamate cycle between glia and neurons [35]. Existing evidence suggests that the aspartate derived by catabolism of NAA in the brain may not be used for the re-synthesis of NAA [22]. Studies demonstrated rapid transamination of doubly labelled (^3^H and ^15^N) aspartate from [^3^H_2_^15^N]NAA, in which the amino group was transferred to glutamate, in the absence of ^15^N-NAA production. However, Miller et al. [22] could not follow the carbon skeleton of NAA since the nitrogen was labelled, not the carbon atoms. Our results, using a ^13^carbon labelled precursor ([1,6-^13^C]glucose), demonstrate that aspartate is not released to the medium by oligodendrocytes in culture, disproving the hypothesis of aspartate recycling between neurons and oligodendrocytes [39]. What we observed was an increase in aspartate content in the oligodendrocytes but the % ^13^C labelling remained unchanged. It is likely that aspartate is converted to oxaloacetate and further to malate in the cytosol, steps in the energy producing malate-aspartate shuttle for transporting reducing equivalents from NADH from glycolysis into the mitochondria [35]. The malate–aspartate shuttle is tightly coupled to glycolysis in that reducing equivalents are transported into the mitochondria for oxidative phosphorylation. Therefore, ATP is produced and NAD, which is necessary for glycolysis, is re-generated in the cytosol. The increased glucose consumption and lactate production observed in the presence of NAA in the present study is indicative of an increased aerobic glycolysis (Warburg effect) also seen in cancer cells. In this context it is noteworthy that exogenously applied NAA has been found to promote growth in several cancer cell lines [41]. A potential explanation consists in a more efficient NAD regeneration due to an increase in the capacity of the malate–aspartate shuttle due to aspartate generation from NAA. Entry of aspartate from NAA into the TCA cycle appears to be slow due to the expected dilution of ^13^C labelling of compounds derived from this cycle, and consequently % enrichment, was not decreased after 8 h of incubation. Aspartate labelling would be expected to increase together with glutamate and glutamine at 8 h, but this was not observed. Indeed, % ^13^C labelling of aspartate was unchanged, indicating that aspartate synthesis from NAA took place in a restricted compartment, which we hypothesise to be the malate-aspartate shuttle. However, after 24 h incubation with NAA the labelling of glutamate and glutamine was not found to be different from the one observed in cells cultured in the absence of NAA, indicating that aspartate was participating in the TCA cycle. This observation may also be related to the slow turnover of NAA detected in vivo. The reported turnover rates of NAA C6 and C3 in rat brain range between 0.7 ± 0.1 and 0.6 ± 0.1 µmol/(g h) with the time constants 14 ± 2 and 13 ± 2 h, respectively, with an estimated pool size of 8 µmol/g [42]. These results suggest that complete label turnover of NAA from glucose occurs in approximately 70 h. This is in agreement with a slow incorporation of aspartate into the oligodendrocyte TCA cycle in order to maintain homeostasis between synthesis and degradation. A potential explanation for the increase in % ^13^C labelling in glutamate and glutamine is a decrease in the amount of ^13^C labelled citrate leaving the TCA cycle as the acetyl CoA content in the cytosol is increased due to the catabolism of NAA. The reduced leakage of labelled substance is expected to cause an overall increase in labelling.

Percent ^13^C labelling of glutamate and glutamine were increased in the presence of NAA. This may be caused by a decrease in ^13^C labelled citrate efflux from the TCA cycle to the cytosol. Citrate is the most common precursor for acetyl CoA production in the cytosol, and also acts as a substrate for lipid synthesis. High levels of NAA in the cytosol of oligodendrocytes incubated with NAA could support acetyl CoA production from acetate generated by hydrolysis of NAA. This is a possibility since ACSS2, the gene for the nuclear-cytosolic form of acyl-CoA synthetase short-chain family member, is expressed in oligodendrocytes to a high extent as reported in transcriptomics studies of the mouse brain [43, 44]. This would decrease the need for citrate catabolism for fatty acid synthesis. In contrast to the increased enrichment of glutamate and glutamine, no differences in the labelling of citrate inside the cells and in the medium were detected. This indicates that the majority of citrate was produced in a separate compartment. The notion of compartmentation is also supported by the much larger % ^13^C enrichment in citrate in the medium compared to that of intracellular metabolites.

It is unlikely that the acetate moiety of NAA released by ASPA entered the mitochondria and was converted to acetyl CoA since this would have resulted in decreased ^13^C labelling of the metabolites, and this was not observed. This is in agreement with a report that oligodendrocytes do not have the mitochondrial enzyme AceCS2 [4]. However, in the mitochondria of oligodendrocytes in culture, we have shown that acetate from the medium is indeed converted to acetyl CoA [30]. As previously mentioned, the most likely fate of the resulting acetyl CoA is its entry into lipid synthesis [6].

### Tri-Cellular Compartmentation of NAA Metabolism?

**Fig. 6:**
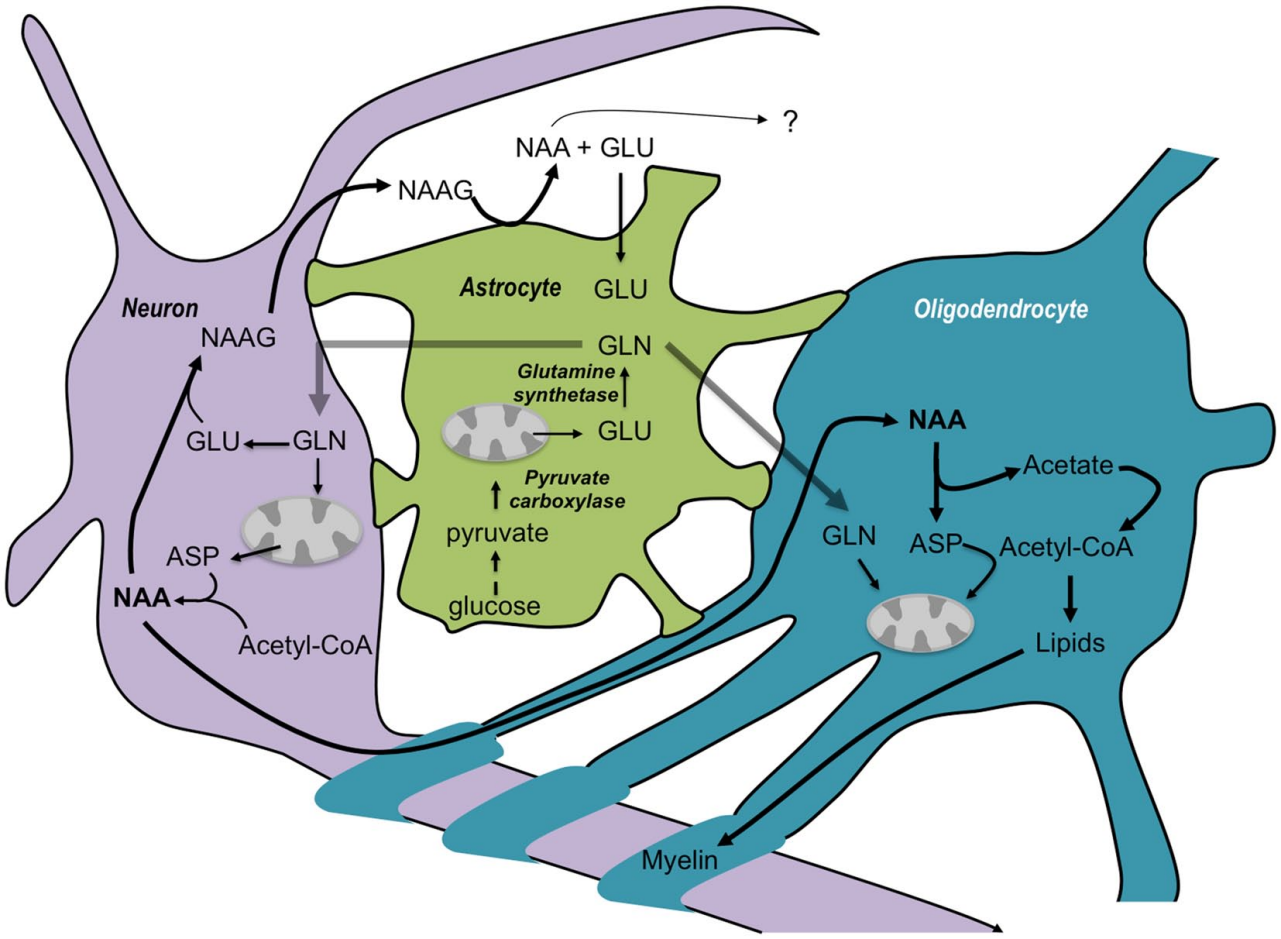
Schematic overview of the metabolic interactions involving *N*-acetyl aspartate (NAA) and *N*-acetyl aspartate glutamate (NAAG) between neurons, oligodendrocytes and astrocytes. Glucose from the blood is taken up by neurons, astrocytes and oligodendrocytes and can be metabolized via glycolysis giving rise to pyruvate formation. Pyruvate can be carboxylated in astrocytes via pyruvate carboxylase and glutamine (GLN) can be formed eventually (for details see [26]). In brain, the predominant cell type for NAA synthesis is neurons that synthesize it from aspartate (ASP) and Acetyl-CoA. NAAG is also synthesized in neurons and converted to NAA in astrocytes. NAA is taken up and metabolized by oligodendrocytes. The acetate moiety produced by hydrolysis of NAA does not enter mitochondrial metabolism in the form of Acetyl-CoA it is used for lipid synthesis. Aspartate is not released to the medium by oligodendrocytes in amounts detectable by our methods. We propose that aspartate released from NAA joins the cytosolic aspartate pool rapidly and takes part in the malate–aspartate shuttle, which transports reducing equivalents from glycolysis into the mitochondria for ATP production and enters the tricarboxylic acid cycle at a slow rate

Tri-cellular compartmentation is necessary for catabolism of NAAG but not for NAA. For the latter, interactions between neurons and oligodendrocytes appear to be sufficient. However, it is important to note that aspartate production in neurons is only possible with the help of glutamine from other cells. Neurons do not express pyruvate carboxylase, the anaplerotic enzyme in the brain [15] and, thus, cannot produce “de novo” aspartate. In order to export NAA, neurons have to import glutamine from external sources [45]. Aspartate is synthesized in the TCA cycle as a result of multiple conversion steps involving glutamate and oxaloacetate. So far only astrocytes are known to synthesise glutamine “de novo” and to release it to the medium [35]. We have recently demonstrated that oligodendrocytes are capable of anaplerosis [32] and therefore meet one of the requirements for supporting neurons. However, it remains unknown whether they are capable of exporting glutamine and, so, support transfer of NAA. Our present findings indicate a tri-cellular compartmentation of NAA metabolism, which starts with astrocytes releasing glutamine, which is taken by neurons, and converted into NAA. Subsequently, NAA released into the extracellular space is taken up and metabolized by oligodendrocytes. Figure 6 summarizes the findings described in the present work. A similar multicellular metabolism of NAA has been proposed depicting some of the metabolite trafficking between various cell types in the brain associated with NAA synthesis and breakdown [4, 46]. The authors stressed that the slow rate of NAA synthesis in neurons and breakdown and utilization in oligodendrocytes is suggestive of non-energy derivation roles in lipid synthesis and protein acetylation reactions under normal conditions, with a shift to much more rapid metabolism in response to injury or disease. The rate of synthesis and breakdown would also be much greater during postnatal myelination than in the normal adult brain and the oligodendrocyte cultures used in the present study might reflect this.

## Conclusion

In addition to the results from a previous study [47], this study provides new evidence that cultured oligodendrocytes hydrolyse NAA. The resulting aspartate and the acetate moieties remain within the cells. As the acetate is not incorporated into TCA cycle intermediates, it is likely to be used in lipid synthesis. The aspartate entity is likely to affect the malate–aspartate shuttle, glycolysis and energy production.
